# High-Resolution Respirometry for Simultaneous Measurement of Oxygen and Hydrogen Peroxide Fluxes in Permeabilized Cells, Tissue Homogenate and Isolated Mitochondria

**DOI:** 10.3390/biom5031319

**Published:** 2015-06-29

**Authors:** Marina Makrecka-Kuka, Gerhard Krumschnabel, Erich Gnaiger

**Affiliations:** 1 Laboratory of Pharmaceutical Pharmacology, Latvian Institute of Organic Synthesis, Riga LV1006, Latvia; E-Mail: makrecka@biomed.lu.lv; 2 OROBOROS INSTRUMENTS, Innsbruck 6020, Austria; E-Mail: Gerhard.Krumschnabel@oroboros.at; 3 Daniel Swarovski Research Laboratory, Department of Visceral, Transplant and Thoracic Surgery, Medical University of Innsbruck, Innsbruck 6020, Austria

**Keywords:** high-resolution respirometry, H_2_O_2_ flux, Amplex Red, HEK 293T, mouse brain homogenate, mouse cardiac mitochondria

## Abstract

Whereas mitochondria are well established as the source of ATP in oxidative phosphorylation (OXPHOS), it is debated if they are also the major cellular sources of reactive oxygen species (ROS). Here we describe the novel approach of combining high-resolution respirometry and fluorometric measurement of hydrogen peroxide (H_2_O_2_) production, applied to mitochondrial preparations (permeabilized cells, tissue homogenate, isolated mitochondria). The widely used H_2_O_2_ probe Amplex Red inhibited respiration in intact and permeabilized cells and should not be applied at concentrations above 10 µM. H_2_O_2_ fluxes were generally less than 1% of oxygen fluxes in physiological substrate and coupling states, specifically in permeabilized cells. H_2_O_2_ flux was consistently highest in the Complex II-linked LEAK state, reduced with CI&II-linked convergent electron flow and in mitochondria respiring at OXPHOS capacity, and were further diminished in noncoupled mitochondria respiring at electron transfer system capacity. Simultaneous measurement of mitochondrial respiration and H_2_O_2_ flux requires careful optimization of assay conditions and reveals information on mitochondrial function beyond separate analysis of ROS production.

## 1. Introduction

Mitochondrial reactive oxygen species (ROS) production contributes to both physiological and pathological processes and is essential in cell life and death decisions [1]. At low concentrations ROS promote the adaptation of cells to stress conditions by the regulation of oxidative metabolism, cellular differentiation and autophagy, thus supporting cell survival. Cellular concentrations of ROS are tightly regulated by cellular antioxidant defense systems. When the cellular antioxidant capacity is overwhelmed, ROS concentrations may increase dramatically and the resulting oxidative stress can cause substantial cell damage and ultimately cell death. Hence, both oxidative stress originating from mitochondrial activity and mitochondrial dysfunction ensuing from related oxidative damage have been shown to play important roles in aging and the pathogenesis of various disease states such as ischemia, neurodegeneration, diabetes and atherosclerosis (reviewed in [2,3,4]).

The magnitude of mitochondrial (mt) ROS production depends on the tissue type, the substrates metabolized, and the site of the mitochondrial electron transfer system (ETS) involved [5]. For the ETS it is quite generally agreed that Complexes I and III, but also the electron-transferring flavoprotein and glycerophosphate dehydrogenase complexes are the main sites of ROS production, particularly under conditions of high mt-membrane potential (reviewed in [6,7]). The primary chemical species of ROS produced by mitochondrial activity appears to be the superoxide anion, most of which is immediately converted to H_2_O_2_ by mitochondrial superoxide dismutase (MnSOD). ROS formation, therefore, can be detected with probes sensitive to H_2_O_2_. Hydrogen peroxide is comparatively stable and, due to its membrane permeability, accessible to such probes. Most of the studies investigating mtROS formation have been performed on isolated mitochondria, as methods employed in animals or cultured cells are considered to not provide accurate and quantitative results [8]. It is important, however, to relate these measurements to ROS production of mitochondria within their physiological microenvironment of the cell under actual *in vivo* conditions [9]. Furthermore, measurements of ROS formation are typically conducted in fluorometer cuvettes using buffers optimized for this purpose and generally not identical with the medium applied for determination of mitochondrial respiratory activity. This makes it impossible to accurately correlate ROS formation and mitochondrial energetics under identical conditions imposing a major uncertainty in fluorometric experiments. Continuous measurements of mitochondrial ROS production reported so far were almost always restricted to rather limited time periods of no more than 15 min [10,11,12,13,14] during which it is hardly possible to accurately evaluate multiple mitochondrial substrate and coupling states.

To overcome some of these limitations, we have recently characterized experimental and technical conditions required for the simultaneous determination of mitochondrial oxygen and H_2_O_2_ fluxes using the OROBOROS O2k-Fluorometer based on the Oxygraph-2k for high-resolution respirometry and the O2k-Fluo LED2-Module for the detection of H_2_O_2_ by Amplex^®^ UltraRed [15]. In the present study we extended this approach to show applications for investigating H_2_O_2_ flux in permeabilized HEK 293T cells, mouse brain homogenate, and isolated mouse heart mitochondria as experimental models, with application of substrate-uncoupler-inhibitor titration (SUIT) protocols to interrogate sequentially different substrate and coupling states (Table 1).

**Table 1:** Definitions of Substrate States and Coupling States used to Characterize Mitochondrial Energetics.

Abbreviation	Definition
**CI-linked**	The Complex I-linked substrate state is induced in mt-preparations by addition of NADH-generating substrates.
**CII-linked**	The Complex II-linked substrate state is induced in mt-preparations by addition of succinate and rotenone (Complex I inhibitor).
**CI&II-linked**	The Complex I&II-linked substrate state is induced in mt-preparations by addition of NADH-generating substrates (CI-linked) in combination with succinate (CII-linked). This physiological substrate combination is required for partial reconstitution of TCA cycle function and convergent electron-input into the Q-junction to compensate for metabolite depletion into the incubation medium. An additive effect of convergent CI&II-linked electron flow is observed in most types of mitochondria.
***R***	In the intact cell, ROUTINE respiration or ROUTINE activity in the physiological coupling state *R* is controlled by cellular energy demand, energy turnover and the degree of coupling to phosphorylation of ADP (intrinsic uncoupling and pathological dyscoupling; [16]).
***L***	LEAK respiration or LEAK oxygen flux compensating for proton leak, proton slip, cation cycling and electron leak, is a dissipative component of respiration which is not available for performing biochemical work and thus related to heat production. LEAK respiration is measured in state *L*, in the presence of reducing substrate(s), but absence of ADP (theoretically, absence of inorganic phosphate presents an alternative), or after enzymatic inhibition of the phosphorylation system. In this non-phosphorylating resting state, the electrochemical proton gradient is increased to a maximum, exerting feedback control by depressing oxygen flux to a level determined mainly by the proton leak and the H^+^/O_2_ ratio [17].
***P***	OXPHOS capacity is the respiratory capacity of mitochondria in the ADP-activated state of oxidative phosphorylation, at saturating concentrations of ADP, inorganic phosphate, oxygen, and defined reduced substrates [17]. It thus differs from State 3 respiration which is respiration of isolated coupled mitochondria in the presence of high ADP and Pi concentrations [18]. ADP concentrations applied in State 3 are not necessarily saturating, whereas OXPHOS capacity is measured at saturating concentrations of ADP and Pi.
***E***	The mitochondrial electron transfer system (ETS) transfers electrons from externally supplied reduced substrates to oxygen. It consists of the membrane-bound ETS (mETS) with enzyme complexes located in the inner mt-membrane, mt-matrix dehydrogenases generating NADH, and the transport systems involved in metabolite exchange across the mt-membranes [19]. ETS capacity is max. O_2_ flux at optimum uncoupler concentration.

## 2. Results and Discussion

### 2.1. Effect of Amplex Red on Respiration of Intact and Permeabilized HEK 293T Cells

**Figure 1:**
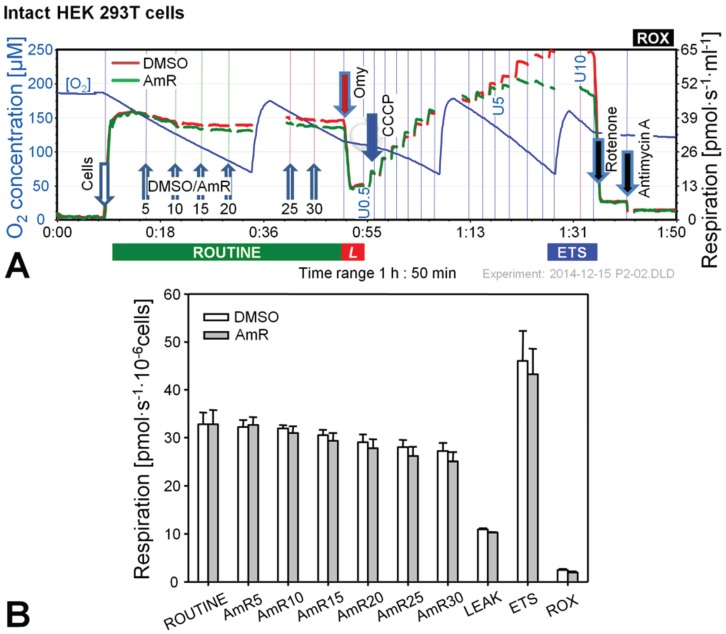
Effect of Amplex UltraRed (AmR) on respiration of intact HEK 293T cells. (**A**) Representative respiratory experiment with AmR or carrier DMSO titrated in the ROUTINE state. Oxygen concentration (blue plot; left *Y*-axis [µM]) is shown for one chamber, whereas oxygen fluxes per chamber volume (red and green plots; right *Y*-axis [pmol·s^−1^·mL^−1^]) are depicted for both O2k-chambers operated simultaneously. The horizontal bar denotes the respiratory states, ROUTINE; LEAK state, *L*; progressively uncoupled states in which ETS capacity is reached at maximum flux; and residual oxygen consumption, ROX. Numbers indicate final AmR concentrations [µM]; U0.5, U5 and U10 indicate final uncoupler concentrations [µM], added in 0.5 µM steps between 0.5 and 5 µM and 1 µM steps between 5 and 10 µM. Discontinuities of the plots are due to removal of sections with artifacts arising from titrations or re-oxygenations; (**B**) Oxygen flow expressed as respiration per million cells [pmol·s^−1^·10^−6^ cells], mean ± SE of *N* = 3–5 independent cultures, each measured in duplicate (*n* = 2).

**Figure 2:**
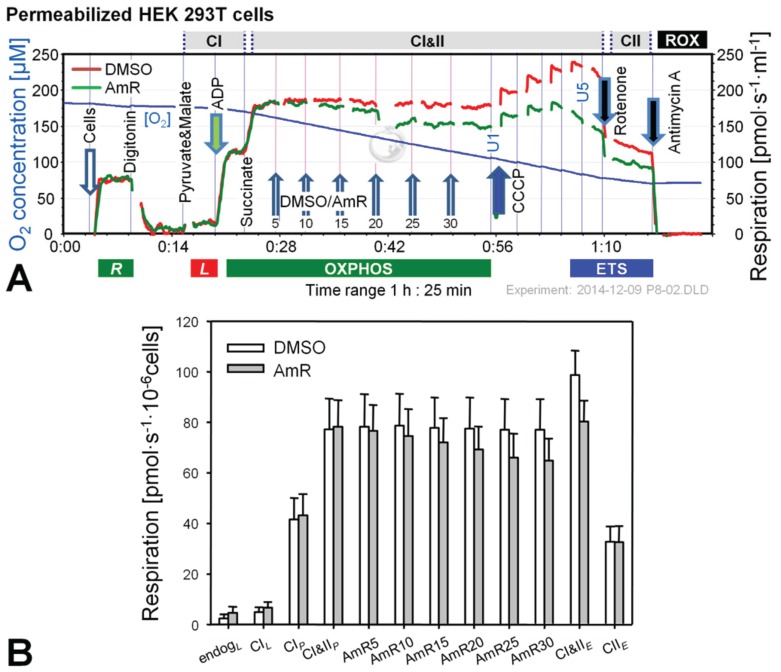
Effect of AmR on respiration of permeabilized HEK 293T cells. (**A**) Representative experiment with AmR (green plot) or carrier DMSO (red plot) titrated in the CI&II-linked OXPHOS state. Substrate states and coupling states are shown by horizontal bars. Numbers indicate final Amp concentrations [µM]; U1 and U5 indicate final uncoupler concentrations [µM] added in 1 µM steps; (**B**) Oxygen flow [pmol∙s^−1^·10^−6^ cells], mean ± SE of *N* = 5 independent cultures, each measured in duplicate (*n* = 2).

The use of Amplex Red or Amplex UltraRed (AmR) at concentrations up to 50 µM is suggested by commercial suppliers for the determination of H_2_O_2_ production, but much lower concentrations down to 1 µM have been applied successfully [12,13,14,20,21]. Since some fluorescence probes, e.g., mitochondrial membrane potential sensitive dyes (safranin, rhodamine), inhibit mitochondrial respiration [22,23], we considered it advisable to check for such undesired effects and to evaluate the optimal concentration of AmR prior to the actual experimental series. The dose-dependent effect of AmR on mitochondrial respiration is shown in Figure 1 and Figure 2 for intact and permeabilized HEK 293T cells.

In the experiments with intact cells titrations with AmR were conducted in the ROUTINE state of respiration. ROUTINE respiration was not completely stable in controls for the duration of the carrier titrations (DMSO) which lasted approximately 35 min. Titration of AmR caused a slight but non-significant further reduction of ROUTINE respiration by up to 8% at 30 µM compared to time-matched controls (Figure 1B). Subsequent addition of oligomycin induced an immediate inhibition of respiration, and LEAK respiration was indistinguishable between both groups. To obtain a measure for ETS, *i.e.*, the maximal capacity of the electron transfer system under the conditions examined, a step-by-step titration with uncoupler CCCP was performed showing that AmR caused a slight but variable, on average insignificant reduction of ETS capacity. Residual oxygen consumption, ROX, obtained after inhibition of Complexes I and III by rotenone (Rot) and antimycin A (Ama), respectively, was identical in controls and AmR-treated cells. Taken together, these results suggest that AmR may be used at concentrations up to 30 µM to determine H_2_O_2_ production in intact HEK 293T cells with minor side effects on respiration. Exposure of these cells to 20 µM AmR for more than 45 min, however, caused about 13% inhibition of ROUTINE respiration [24], suggesting that AmR concentrations and exposure time should be limited. The inhibitory effect depends on the medium used. Since the sensitivity of the AmR assay was rather low in DMEM, we applied Dulbecco’s phosphate-buffered saline, in which case excellent assay sensitivity was associated with seriously compromised respiration, both with (up to 50% inhibition) and without AmR (up to 20% inhibition). A brief literature survey indicates that AmR concentrations and media applied are highly variable, ranging from 1 µM AmR in PBS for the study of permeabilized C2C12 myoblasts and myotubes [25], 10 µM AmR in MiR05 for primary human skeletal myotubes [26], to 50 µM AmR in various phosphate-buffered or bicarbonate-buffered saline media investigating N27 cells [27], A549 lung epithelial cells [20], HaCaT keratinocytes [28], or HEK 293T cells [29], or mitochondria prepared from H9c2 rat cardiac myocytes [29] or from HepG2 cells [30]. In the absence of adequate controls it is not clear to which extent these treatments might have affected the results on H_2_O_2_ production. Therefore, careful optimization is required both with regard to AmR concentrations and the incubation media for quality control of measurements on H_2_O_2_ production in cells.

Experiments with permeabilized cells were performed in MiR05 [31] and AmR was titrated in the CI&II-linked OXPHOS state, *i.e.*, in the presence of pyruvate, malate and succinate at saturating concentration of ADP (Figure 2). Respiration in controls was not affected by the carrier DMSO. In contrast, AmR inhibited CI&II-linked OXPHOS capacity in a dose-dependent manner, resulting in 15% ± 7% inhibition at 30 µM (mean ± SD, *N* = 5). Similarly, CI&II-linked ETS capacity was reduced by 19% ± 8%, with a shift to a lower optimum uncoupler concentration compared to controls. CII-linked ETS capacity was not affected, possibly indicating that AmR inhibition occurred at CI, which is highly sensitive to agents damaging mitochondria [22,32,33]. In order to minimize such side effects, the AmR concentration was reduced to 10 µM for experiments with permeabilized cells.

### 2.2. O_2_ and H_2_O_2_ Flow in Permeabilized HEK 293T Cells: Dependence on Substrate and Coupling State

**Figure 3:**
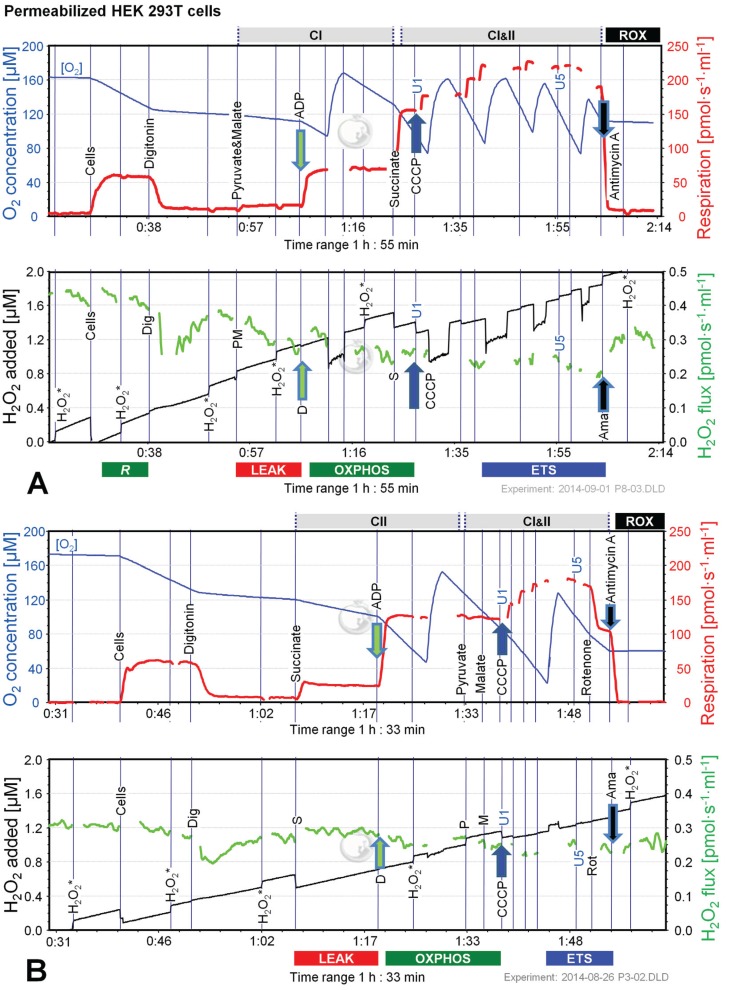
Combined determination of oxygen consumption and H_2_O_2_ flux by O2k-Fluorometry in permeabilized HEK 293T cells. (**A**) Respiration and fluorescence changes using P and M as initial substrates; (**B**) Respiration and fluorescence changes using succinate (S) as initial substrate. Respirometric measurements are shown in the upper panels as described in Figure 1. U1 and U5 indicate final uncoupler concentrations [µM] added in 1 µM steps. In the lower panels the black plots show the fluorescence signal. H_2_O_2_* indicates titrations of 0.1 µM H_2_O_2_ for calibration, to convert the fluorescence signal to an equivalent H_2_O_2_ concentration (left *Y*-axis [µM]). Plots are shown on the basis of the first calibration with H_2_O_2_. The positive time derivative yields the volume-specific H_2_O_2_ flux shown as the green plots (right *Y*-axis [pmol·s^−1^·mL^−1^]).

**Figure 4:**
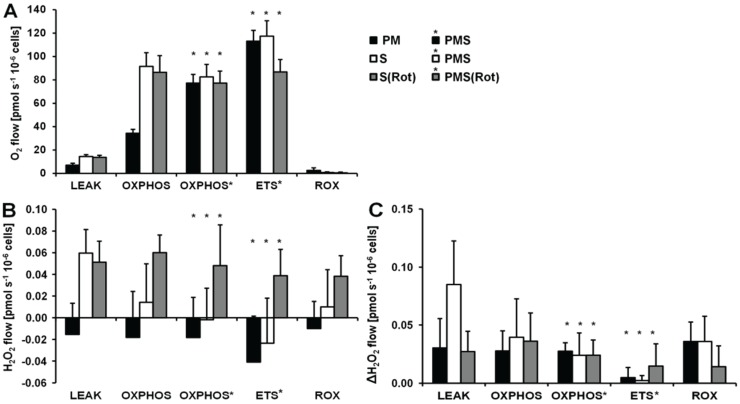
Respiration and H_2_O_2_ flow in permeabilized HEK 293T cells in SUIT protocols using PM (black bars), S (white bars) and S(Rot) (grey bars) as initial substrates. (**A**) Oxygen flow; (**B**) H_2_O_2_ flow corrected for the background slope determined in the absence of cells; (**C**) H_2_O_2_ flow corrected for the lowest observed slope. The fluorescence signals were calibrated using the H_2_O_2_ titrations at the corresponding state (Figure 3). Bars are means ± SD of four independent cultures measured in duplicates.

O_2_ and H_2_O_2_ fluxes were determined in permeabilized HEK 293T cells in a sequence of respiratory substrate and coupling states using pyruvate&malate (PM; CI), pyruvate&malate&succinate (PMS; CI&II) or succinate with Rot (S(Rot)) as respiratory substrates (Figure 3). Results are summarized in Figure 4 and Table 2. The H_2_O_2_/O flux ratio is frequently applied to evaluate the relative importance of H_2_O_2_ production at different respiratory states [34,35].

After permeabilization of the cell membranes with digitonin, addition of PM as substrates supporting CI-linked LEAK respiration (CI*_L_*) induced a moderate increase in respiration (Figure 3A). ADP added at a saturating concentration stimulated respiration about 5 times (CI-linked OXPHOS capacity, CI*_P_*). Succinate induced convergent CI&II-linked OXPHOS (CI&II*_P_*), at a 2.2-fold higher level compared to CI*_P_*. CCCP at optimum concentration elevated respiration further, showing a significant apparent excess ETS capacity in these cells. Subsequently Rot and Ama were added to obtain ROX. Despite these dramatic differences in oxygen fluxes in different respiratory states, alteration in H_2_O_2_ fluxes were comparatively small. When total observed H_2_O_2_ flux was corrected for the background chemical flux obtained in the absence of cells, the net fluxes were negative. This may indicate the ROS scavenging capacity introduced with the cells, as will be discussed below. Thus, even the most pronounced relative change in H_2_O_2_ flux induced by inhibition of CIII (Ama) after ETS was close to the chemical background. As an alternative approach, the lowest flux detected in each experimental run was subtracted from H_2_O_2_ flux in each respiratory state, expressing H_2_O_2_ flux not as an absolute metabolic flux but as a difference, ΔH_2_O_2_ flux (Figure 4C). The lowest H_2_O_2_ flux was observed in fully uncoupled cells in almost all cases.

**Table 2:** H_2_O_2_/O flux ratios [%] as a function of coupling and substrate states in permeabilized HEK cells and mouse brain homogenate.

Substrate	LEAK	OXPHOS	PMS OXPHOS	PMS ETS	ROX
	**Permeabilized HEK cells**				
S	0.85 ± 0.34	0.03 ± 0.08	na	na	7.2 ± 22.8
S(Rot)	0.81 ± 0.55	0.14 ± 0.07	0.16 ± 0.07	0.10 ± 0.7	7.9 ± 3.4
	**Mouse brain homogenate**				
PM	1.2 ± 0.6	0.06 ± 0.13	na	na	60 ± 13
S	6.3 ± 0.2	0.05 ± 0.13	0.05 ± 0.09	na	26 ± 6
S(Rot)	1.5 ± 0.2	0.51 ± 0.08	1.34 ± 0.09	1.24 ± 0.06	33 ± 8

The H_2_O_2_/O flux ratio was calculated as H_2_O_2_ flux/(0.5 O_2_ flux). Means ± SD of 4 independent cultures or 3 animals. na—not applicable.

LEAK respiration supported by succinate (CII*_L_*) was about three times higher than *L* supported by PM (CI*_L_*) and was associated with much higher H_2_O_2_ flux in the absence of rotenone. ADP induced a 6.7-fold increase in respiration (CII*_P_*) and a concurrent pronounced reduction in H_2_O_2_ flux (Figure 3B). Surprisingly, addition of pyruvate did not stimulate respiration, and malate caused a slight inhibition, such that CI&II*_P_* respiration was slightly reduced compared to CII*_P_*, whereas H_2_O_2_ flux remained constant. The fact that rotenone was not required to obtain a high CII-linked OXPHOS capacity is consistent with a high malic enzyme activity in these cells [36]. Thus, oxaloacetate does not accumulate to concentrations which inhibit succinate dehydrogenase, but pyruvate and further acetyl-CoA are formed from malate, supporting the utilization of oxaloacetate in the citrate synthase reaction [19,37]. Formation of NADH and its utilization by CI, therefore, proceeded in the CII-linked OXPHOS state, and CI&II-linked and CII-linked OXPHOS capacities were not different (Figure 4). However, CI&II*_P_* was limited by the capacity of the phosphorylation system, as shown by the increased ETS capacity with PMS (CI&II*_E_*) which was higher than CII*_E_*, indicating the additive effect of convergent electron flow from CI&II to the Q-junction in these cells [38]. CCCP titration reduced the H_2_O_2_ flux to a level slightly below baseline. As observed in the previous protocol, Ama induced an increase in H_2_O_2_ flux (Figure 4).

Respiration and H_2_O_2_ flux in the LEAK state were similar with S(Rot) and S alone (Figure 4). In many other cells and tissues (e.g., rat brain homogenate as shown below), Rot causes a significant reduction of H_2_O_2_ production observed in the CII-linked LEAK state due to inhibition of reversed electron flow to CI [39]. The absence of such an effect is consistent with the effect of malic enzyme on respiration [19]. In contrast to the presence of S alone, addition of ADP to S(Rot) exposed cells did not decrease the H_2_O_2_ flux. The effects of PM and CCCP were relatively small, and the addition of Ama did not stimulate ROS production, in contrast to results obtained with the other protocols (Figure 4).

Taken together, in permeabilized HEK 293T cells the highest H_2_O_2_ flux was observed in the CII-linked LEAK state, amounting to about 0.8% of oxygen flux (Table 2). In comparison, CI-linked H_2_O_2_ flux appears negligible, while inhibition of CI increased H_2_O_2_ production independent of respiratory state. H_2_O_2_ flux in permeabilized HEK 293T cells was extremely low under the presently investigated conditions, accounting for less than 0.2% of O_2_ flux in the OXPHOS and ETS states (Table 2).

### 2.3. O_2_ and H_2_O_2_ Flow in Permeabilized HEK 293T Cells: Dependence on Cell Density

**Figure 5:**
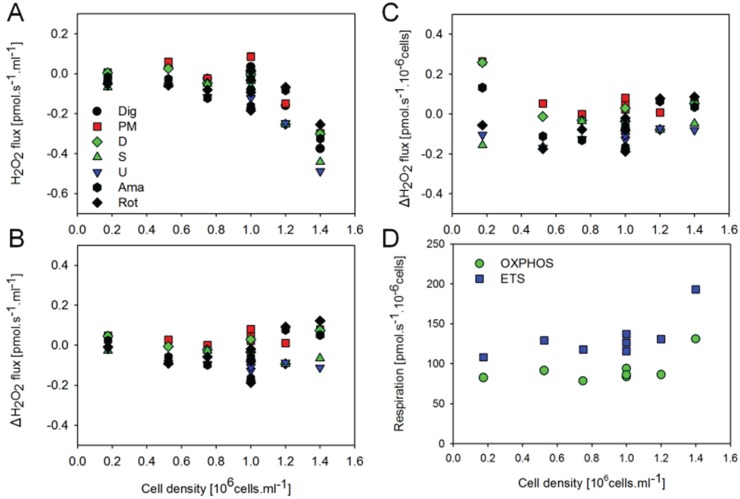
Effect of cell concentration in the O2k-chamber on H_2_O_2_ flux and respiration of permeabilized HEK 293T cells. (**A**) H_2_O_2_ flux (per O2k-chamber volume) corrected for the slope determined in the absence of cells; (**B**) H_2_O_2_ flux (per O2k-chamber volume) corrected for the slope after addition of digitonin; (**C**) H_2_O_2_ flow (per million cells) corrected for the slope after addition of digitonin; (**D**) Respiratory flow (per million cells). H_2_O_2_ fluxes were calculated according to calibrations after addition of digitonin.

Given that estimated H_2_O_2_ fluxes were close to or even below background observed in the absence of cells, the sensitivity of the AmR assay may present a limiting factor at the experimental cell density. By increasing the cell density in the respiration chamber, however, the slopes of the fluorescence signal corrected for the background determined in the absence of cells actually displayed an inverse relation to cell density (Figure 5A). In contrast, when corrected for the slope observed in the presence of digitonin permeabilized cells without substrates added, net H_2_O_2_ fluxes were largely independent of cell density (Figure 5B). Importantly, cell-specific respiration and net H_2_O_2_ flow (per million cells) were independent of cell density (Figure 5C,D). A tentative explanation for these observations is that by increasing the number of cells in the chamber we also enhance the total ROS scavenging capacity associated with the cellular antioxidant systems. In addition, the optical properties are affected by cell density, as shown by the step change of the fluorescence signal upon injection of cells, and by the change of sensitivity when comparing calibrations before and after addition of cells, and after titration of digitonin. For example, sensitivity declined from 0.282 ± 0.019 V/µM to 0.260 ± 0.019 V/µM after addition of cells (*n* = 8 experiments), from 0.249 ± 0.033 V/µM to 0.179 ± 0.018 V/µM after adding brain tissue homogenate (*n* = 8 experiments), and from 0.262 V/µM to 0.250 V/µM after injection of mitochondria (means of two experiments). Whereas calibration of the fluorescence signal in the absence of cells is required to obtain the apparent background flux, H_2_O_2_ calibrations are required in the presence of cells and under various respiratory states (Figure 3).

### 2.4. O_2_ and H_2_O_2_ Flux in Mouse Brain Homogenate: Dependence on Substrate and Coupling State

**Figure 6:**
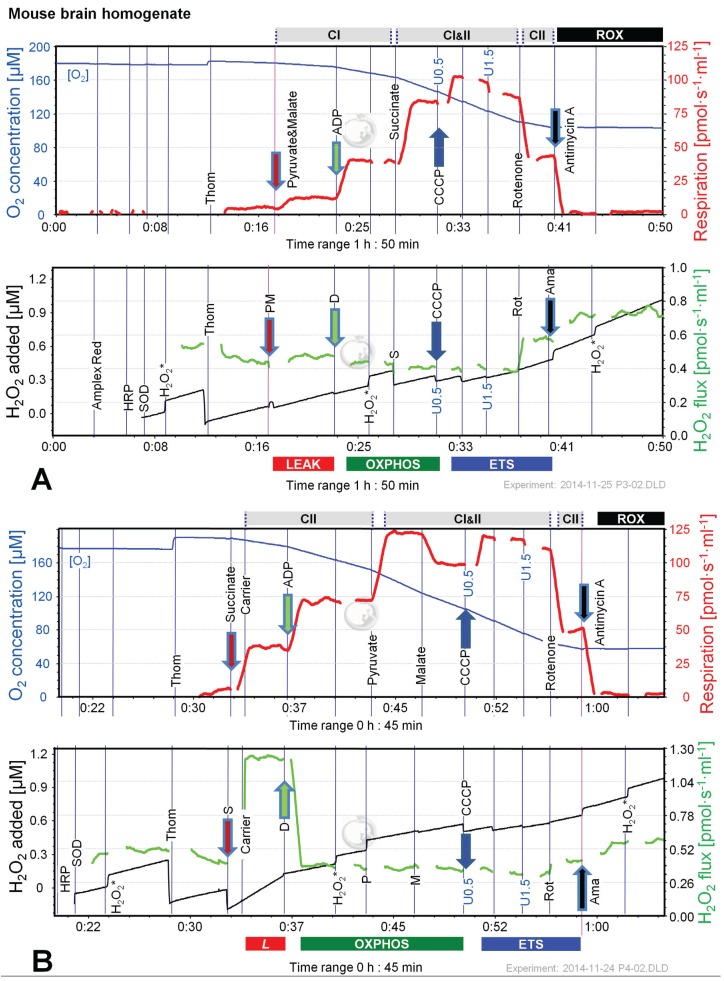

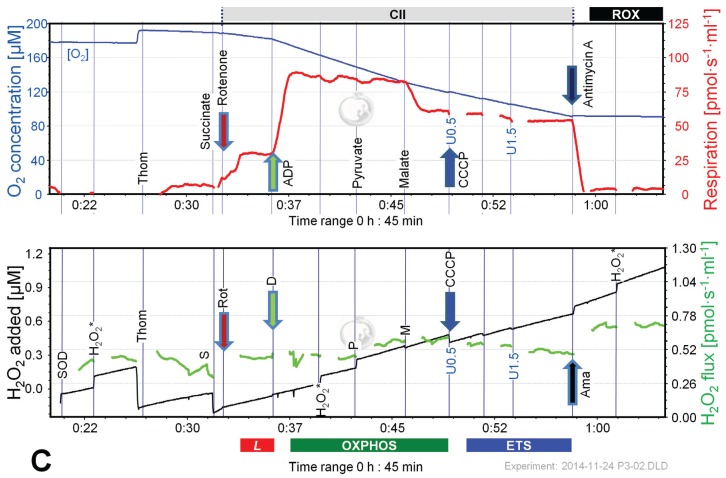
Combined determination of oxygen consumption and H_2_O_2_ flux by O2k-Fluorometry in mouse brain homogenate, using PM (**A**); S (**B**); or S(Rot) as initial substrate (**C**). For details see legend of Figure 3. Carrier denotes ethanol (1 µL), which served as the solvent for rotenone and was titrated in parallel to rotenone.

**Figure 7:**
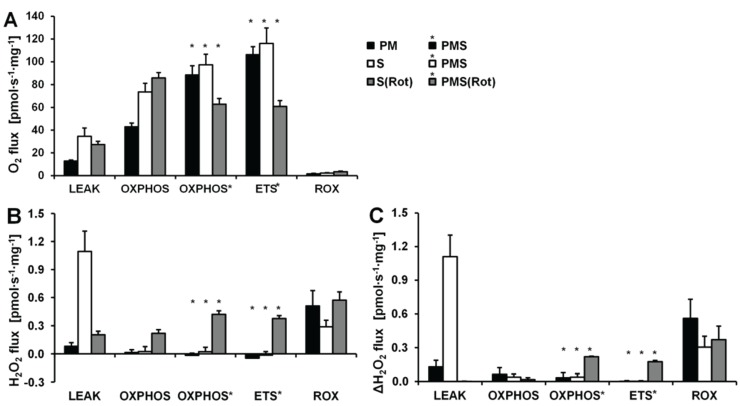
Respiration and H_2_O_2_ flux in mouse brain homogenate in SUIT protocols using PM (black bars), S (white bars) and S(Rot) (grey bars) as initial substrates. (**A**) Oxygen flux. (**B**) H_2_O_2_ flux corrected for the slope determined in the absence of homogenate; (**C**) H_2_O_2_ flux corrected for minimal observed slope. The fluorescence signals were calibrated using the H_2_O_2_ titrations at the corresponding state (Figure 6). Bars are means ± SD of 3 animals, each measured in duplicate.

Figure 6 shows representative experiments of respiration and H_2_O_2_ fluxes in mouse brain homogenate with identical SUIT protocols as applied with permeabilized HEK 293T cells (Figure 3). In contrast to permeabilized HEK 293T cells (Figure 4), CII*_L_* respiration was lower but CII*_P_* was higher with S(Rot) compared to S (Figure 7A). Addition of PM to S induced an increase of respiratory OXPHOS capacity (CI&II*_P_*), despite of the inhibitory effect of malate (Figure 6B). Consistent with results in permeabilized cells, malate caused a significant decrease of CII*_P_* respiration with S(Rot) (Figure 6C). The inhibitory effect of malate on CII-linked respiration is a general feature of TCA cycle control [40].

In contrast to results with permeabilized cells, H_2_O_2_ flux in state CI*_L_* was well above background in brain homogenate (Figure 6A). As in permeabilized HEK 293T cells, the highest H_2_O_2_ flux was observed in the LEAK state with S alone (Figure 6B), and H_2_O_2_ flux was significantly reduced in the OXPHOS and ETS state compared to LEAK (Figure 7B). However, addition of Rot caused a significant reduction of H_2_O_2_ flux, as did addition of ADP (Figure 7B). Compared to CI*_L_*, H_2_O_2_ flux in CII(Rot)*_L_* was 2.5 times higher (Figure 7B). No significant difference in H_2_O_2_ flux was noted between LEAK and OXPHOS states with S(Rot). Surprisingly, addition of PM in state CII(Rot)*_P_* caused an increase of H_2_O_2_ flux, although Complex I was inhibited by rotenone (Figure 6A and Figure 7B). As expected [41], inhibition of CIII by Ama (ROX state) caused an increase in H_2_O_2_ flux in all SUIT protocols (Figure 6 and Figure 7B). H_2_O_2_ flux resulted in similar patterns when corrected for background without homogenate (Figure 7B) or when presented as the difference of flux after subtraction of the minimal observed slope (Figure 7C).

The extraordinarily high H_2_O_2_/O flux ratio of 6% in CII*_L_* without Rot reflects maximal reversed electron transfer under an artificial substrate condition (Table 2). H_2_O_2_/O flux ratios between 0.05% and 1.35% in OXPHOS and ETS states (Table 2) are consistent with values reported for isolated mitochondria [1,11,42]. It is thought that isolation procedures may impact on mitochondrial function (e.g., [43,44]). Isolated mitochondrial preparations may represent a selection for particular mitochondrial subpopulations. This is not the case in tissue homogenate or permeabilized tissue preparations. Tissue homogenization can be achieved without injury of the outer mitochondrial membrane using specifically dedicated instruments such as the PBI tissue shredder [45,46]. A more simple glass Potter homogenizer can be used similarly for homogenate preparation of soft tissues.

Few comparable data are published on permeabilized cells. From data by Kwak *et al.* [26] on permeabilized human skeletal myotubes it can be calculated that about 0.9% of LEAK respiration measured in the presence of a complex substrate mixture was diverted towards H_2_O_2_ production, in close agreement with our result on 0.8% for HEK 293T cells in the presence of succinate (Table 2). In permeabilized yeast cells the H_2_O_2_/O ratio varied between 0.25% and 2% depending on the glucose level supplied and the growth phase investigated [47]. The H_2_O_2_/O flux ratio determined in a mutant *E. coli* strain was 0.35% to 0.6% in different substrate regimes [48].

### 2.5. O_2_ and H_2_O_2_ Flux in Isolated Cardiac Mitochondria: Dependence on Substrate and Coupling State

**Figure 8:**
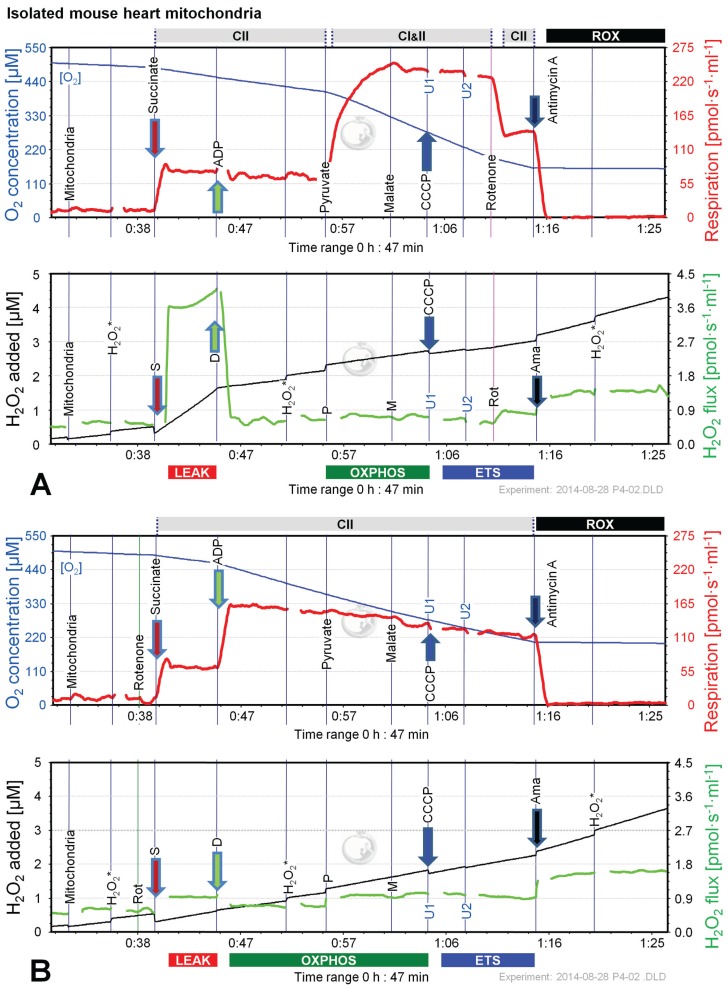
Combined determination of oxygen consumption and H_2_O_2_ production by O2k-Fluorometry in mouse isolated cardiac mitochondria, using S (**A**); or S(Rot) (**B**) as initial substrate. For details see legend of Figure 3.

Since the most pronounced changes of H_2_O_2_ flux in permeabilized cells and brain homogenate were observed in protocols using S and S(Rot), we performed additional experiments applying CII-linked protocols with mitochondria isolated from mouse hearts. Like in mouse brain homogenate, the H_2_O_2_ flux was extremely high in the succinate-supported LEAK state in the absence of Rot, resulting in a H_2_O_2_/O flux ratio of nearly 10%, compared to 1.5% in the presence of Rot (Figure 8). The addition of ADP to S(Rot) stimulated respiration and concurrently reduced H_2_O_2_ flux (Figure 8B). In contrast, addition of ADP to S did not increase respiration, but dramatically diminished H_2_O_2_ flux (Figure 8A). The subsequent addition of P caused a pronounced increase of respiratory OXPHOS capacity, by removing the inhibitory oxaloacetate and restoring CI&II-linked TCA cycle activity [19]. Malate exerted an inhibitory effect in the presence and absence of Rot, similar to results with brain homogenate (Figure 6B,C). Uncoupler did not stimulate respiration beyond OXPHOS capacity, indicating that there is no apparent ETS excess capacity in mouse heart in contrast to mouse brain mitochondria. H_2_O_2_ fluxes were slightly elevated by P in both protocols, largely unresponsive to M, and slightly diminished by uncoupler, while Rot and Ama caused a substantial increase of H_2_O_2_ flux (Figure 8A,B). H_2_O_2_/O flux ratios ranged from 0.04% in ETS in the absence of Rot to 0.9% in OXPHOS and ETS in the presence of Rot, consistent with data reported in the literature [5,49].

## 3. Experimental Section

### 3.1. Chemicals

Dulbecco’s modified eagle medium (DMEM-low glucose, with l-glutamine) was from PAA Laboratories GmbH, Pasching, Austria, fetal bovine serum from Biowest, Nuaillé, France, and penicillin and streptomycin stocks were from Gibco, Vienna, Austria.

Amplex^®^ UltraRed was obtained from Life Technologies. H_2_O_2_, HRP, SOD, substrates, inhibitors and other chemicals were from Sigma-Aldrich, Acros Organics or Invitrogen [50].

### 3.2. High-Resolution Respirometry and O2k-Fluorometry

The Oxygraph-2k (O2k, OROBOROS Instruments, Innsbruck, Austria) was used for measurements of respiration [50] and combined with the Fluorescence-Sensor Green of the O2k-Fluo LED2-Module for H_2_O_2_ measurement. Up to four O2k instruments (eight chambers) were used in parallel. Experiments using tissue homogenate and permeabilized cells were performed in MiR05 (110 mM sucrose, 60 mM K-lactobionate, 0.5 mM EGTA, 3 mM MgCl_2_, 20 mM taurine, 10 mM KH_2_PO_4_, 20 mM HEPES, pH 7.1 at 30 °C, and 0.1% BSA essentially fatty acid free; [31]). Dulbecco’s modified eagle medium supplemented with 10% fetal bovine serum and 50 units/mL penicillin and 50 μg/mL streptomycin was used for measurements on intact cells. All experiments were performed at 37 °C. The medium was reoxygenated when oxygen concentrations reached 80 µM unless otherwise indicated.

### 3.3. Experimental Procedure

Respiration of permeabilized cells and tissue homogenate was determined using substrate-uncoupler-inhibitor titration (SUIT) protocols [50] with modifications. Pyruvate and malate (5 mM and 0.5 mM, respectively) or succinate (10 mM) with or without 0.5 µM Complex I inhibitor rotenone (Rot) were used to determine Complex I (CI) or Complex II (CII) linked LEAK respiration. ADP was added at 2.5 mM final concentration, which was saturating for oxygen flux to obtain OXPHOS capacity. Additional substrates were added sequentially to reconstitute convergent CI&II-linked respiration. Titrations with the uncoupler CCCP (0.5–1 µM steps) were performed to determine electron transfer system (ETS) capacity. Rot, if not already present, and Ama (2.5 µM to inhibit Complex III) were added for determination of residual oxygen consumption (ROX).

Respiration of intact cells was measured applying a coupling control protocol [19]. Up to 300 µL of suspended cells were added to the respiration medium. After stabilization of ROUTINE respiration, the ATP-synthase inhibitor oligomycin (Omy, 2 µg/mL) was added to obtain a measure of LEAK respiration, followed by titration of CCCP to maximum oxygen flux (ETS capacity). Finally, Rot and Ama were added to obtain ROX.

H_2_O_2_ flux was measured simultaneously with respirometry in the O2k-Fluorometer using the H_2_O_2_-sensitive probe Amplex® UltraRed [15]. 10 µM Amplex® UltraRed (AmR), 1 U/mL horse radish peroxidase (HRP) and 5 U/mL superoxide dismutase (SOD) were added to the chamber. The reaction product between AmR and H_2_O_2_, catalyzed by HRP, is fluorescent, similar to resorufin. Calibrations were performed with H_2_O_2_ repeatedly added at 0.1 µM steps as indicated (H_2_O_2_*). Volume-specific H_2_O_2_ fluxes were calculated real-time by the DatLab software (OROBOROS INSTRUMENTS, Innsbruck, Austria) from the positive time derivative of the resorufin signal over time (converted to H_2_O_2_ concentration based on the calibrations with H_2_O_2_). Only the stable portions of the apparent fluxes were selected and artifacts induced by additions of chemicals or re-oxygenations were excluded.

### 3.4. Cell Culture

Human embryonic kidney cells (HEK 293T, ATCC collection code CRL-1573) were cultured in 10 cm^2^ culture dishes in DMEM high glucose medium supplemented with additions as indicated above until approximately 90% confluence was reached. Immediately prior to respirometric assays the cells were washed with PBS, trypsinized and resuspended in MiR05 or DMEM. The final concentration of permeabilized cells in the O2k-chamber was 1.75·10^6^/mL or 1.5–2·10^6^/mL when intact cells were examined. Cells were permeabilized after addition to the respirometer chambers using digitonin at a final concentration of 10 µg/mL. This concentration was evaluated in preliminary experiments to achieve full permeabilization of cells to allow for uninhibited access of substrates and ADP to mitochondria without compromising mitochondrial function [50].

### 3.5. Preparation of Mouse Brain Homogenate

Wild-type C57BL/6 mice (age 2–3 months) were housed under standard conditions (21–23 °C, 12 h light/dark cycle, relative humidity 45%–65%) with unlimited access to food and water. The experimental procedures were performed in accordance with the guidelines of the European Community as well as local laws and policies.

Animals were sacrificed by cervical dislocation, the skull opened with scissors and the brain removed. Brain cortex was dissected and washed in ice-cold MiR05Cr (MiR05 supplemented with 20 mM creatine). The tissue was transferred to a pre-cooled glass Potter homogenizer and homogenized with 10–15 strokes at medium speed. The resulting homogenate was then kept on ice and used for respirometry without further processing. The final concentration of tissue in the O2k-chamber was 1 mg/mL.

### 3.6. Isolation of Mouse Heart Mitochondria

Wild-type C57BL/6 mice were sacrificed by cervical dislocation and the heart was excised and weighed. The heart was washed in ice-cold BIOPS and minced in 1 mL of BIOPS. The tissue was transferred to a pre-cooled glass Potter homogenizer with 2 mL of isolation buffer (225 mM mannitol, 75 mM sucrose, 1 mM EGTA, 2.5 mg/mL BSA) supplemented with Subtilisin (0.5 mg/mL). The tissue was homogenized with 6–8 strokes at medium speed. The resulting homogenate was centrifuged for 10 min at 800× *g*, 4 °C. Then, the supernatant was transferred to a new tube and centrifuged for 10 min at 10,000× *g*, 4 °C. After centrifugation, the supernatant was carefully discarded, the mitochondrial pellet was washed in 2 mL isolation buffer and resuspended in 100 µL of isolation buffer. Isolated heart mitochondria were stored on ice until use. 5 µL of mitochondrial suspension per chamber were used for each measurement.

## 4. Conclusions

SUIT protocols allow a detailed analysis of mitochondrial fitness in permeabilized tissues and cells, tissue homogenates and isolated mitochondria, extended by combining OXPHOS analysis with measurement of hydrogen peroxide production. Combined measurements provide the basis for quality control to avoid experimental artifacts. AmR inhibited respiration of intact and permeabilized cells and should not be applied at concentrations above 10 µM nor during prolonged exposure. The choice of experimental medium is critical and simple media may aggravate the inhibitory effect of AmR. Inhibition of respiration (e.g., by Rot and Ama) exerts an influence on H_2_O_2_ production which is not generally predictable. When increasing the cell density, the cellular ROS scavenging capacity is increased together with the total H_2_O_2_ production, which provides the explanation for our observation that volume-specific H_2_O_2_ production remained constant or even declined with increasing cell density. H_2_O_2_ fluxes were generally less than 1% of oxygen fluxes in physiological substrate and coupling states. In permeabilized cells only net rates are obtained on H_2_O_2_ production escaping the cellular scavenging systems.

## Abbreviations (See also Table 1)

Ama	Antimycin A	Rot	rotenone
AmR	Amplex UltraRed	ROX	residual oxygen consumption
Dig	digitonin	S	succinate
G	glutamate	SOD	superoxide dismutase
HRP	horse radish peroxidase	SUIT	substrate-uncoupler-inhibitor titration
M	malate	Thom	tissue homogenate
P	pyruvate	U	uncoupler
